# Law Pertaining to Dentistry

**Published:** 1876-07

**Authors:** Jno. F. Hartranft, M. S. Quay

**Affiliations:** Governor of Pennsylvania


### LAW PERTAINING TO DENTISTRY.

It is with much gratification that we give place to the following from the June No. of the *Dental Cosmos.*

This is an excellent law, explicit and to the point in all the details. It will certainly exercise a great influence for good on the profession in Pennsylvania.

### AN ACT TO REGULATE THE PRACTICE OF DENTISTRY IN THE STATE OF PENNSYLVANIA.

After considerable effort and many delays, the bill to regulate the practice of dentistry in Pennsylvania has received the signature of the Governor and become a law, in operation from the date thereof. We give the full text of the bill as passed:

An Act to regulate the practice of dentistry and protect the people against empiricism in relation thereto, in the State of Pennsylvania, and providing penalties for the violation of the same.

Section 1. *Be it enacted by the Senate and House of Representatives of the Commonwealth of Pennsylvania, in General Assembly met, and it is hereby enacted by authority of the same,*

That from and after the passage of this act, it shall be unlawful for any person except regularly authorized physicians and surgeons to engage in the practice of dentistry in the State of Pennsylvania, unless said person has graduated and received a diploma from the faculty of a reputable institution where this specialty is taught and chartered under the authority of some one of the United States, or of a foreign government, acknowledged as such, or shall have obtained a certificate from a board of examiners, duly appointed and authorized by the provisions of this act to issue such certificate.

SEC. 2. That the board of examiners shall consist of six practitioners of dentistry, who are of acknowledged ability in the profession. Said board shall be elected by the Pennsylvania State Dental Society at their next annual meeting, as follows: Two shall be elected for one year, two for two years, and two for three years, and each year thereafter two shall be elected to serve for three years, or until their successors are elected. The said board shall have power to fill all vacancies for unexpired terms, and they shall be responsible to said State Dental Society for their acts.

SEC. 3. That it shall be the duty of this board:

*First.* To meet annually at the time and place of meeting of the Pennsylvania State Dental Society, and at such other time and place as the said board shall agree upon, to conduct the examination of applicants. They shall also meet for the same purpose at the call of any four members of said board, at such time and place as may be designated. Thirty days' notice must be given of the meetings by advertising in at least three periodicals, one of them being a dental journal, and all published within this State.

*Second.* To grant a certificate of ability to practice dentistry, which certificate shall be signed by said board and stamped with a suitable seal, to all applicants who undergo a satisfactory examination, and who receive at least four affirmative votes.

*Third.* To keep a book in which shall be registered the names and the qualifications of such, as far as practicable, of all persons who have been granted certificates of ability to practice dentistry under the provisions of this act.

SEC. 4. That the book so kept shall be a book of record, and a transcript from it, certified to by the officer who has it in keeping, with the seal of said board of examiners, shall be evidence in any court of this State.

SEC. 5. That four members of this board shall constitute a quorum for the transaction of business; and should a quorum not be present on any day appointed for their meeting, those present may adjourn from day to day until a quorum is present.

SEC. 6. That any person who shall in violation of this act practice dentistry in the State of Pennsylvania, shall be liable to indictment in the court of quarter sessions of the proper county; and, on conviction, shall be fined not less than fifty, or more than two hundred dollars, *Provided*, That any person so convicted shall not be entitled to any fee for services rendered; and if a fee shall have been paid, the patient, or his or her heirs, may recover the same as debts of like amount are now recoverable by law.

SEC. 7. That all fines collected shall inure to the poor fund of the county in which the prosecution occurs.

SEC. 8. That nothing in this act shall apply to persons who shall have been engaged in the continuous practice of dentistry in this State for three years or over, at the time of or prior to the passage of this act.

SEC, 9. That, to provide a fund to carry out the provisions of the third section of this act, it shall be the duty of the said board of examiners to collect from those who receive the certificate to practice dentistry the sum of thirty ($30) dollars each, of which sum, if there be any remaining after liquidating necessary expenses, the balance shall be paid into the treasury of the said Pennsylvania State Dental Society, to be kept as a fund for the more perfect carrying out of the provisions of this act.

Signed April 17th, 1876. JNO. F. HARTRANFT,
Governor of Pennsylvania.

M. S. QUAY, Secretary.

